# Ambulatory laparoscopic cholecystectomy: Is it safe and cost effective?

**DOI:** 10.4103/0972-9941.51314

**Published:** 2009

**Authors:** Athar Ali, Tabish Chawla, Abid Jamal

**Affiliations:** Department of Surgery, Aga Khan University, Stadium Road, Karachi, Pakistan; 1 OMI Hospital, Karachi, Pakistan

**Keywords:** Ambulatory, cost, laparoscopic cholecystectomy

## Abstract

**BACKGROUND:**

Laparoscopic cholecystectomy (LC) is the most commonly performed minimal invasive surgery. However, practice of its use as an ambulatory surgery in our hospital settings is uncommon.

**OBJECTIVE:**

To evaluate safety and cost effectiveness of LC as an ambulatory day care surgery.

**STUDY DESIGN:**

Quasiexperimental.

**SETTING:**

Department of surgery, Aga Khan University Hospital, Karachi, Pakistan.

**MATERIALS AND METHODS:**

Patients with uncomplicated symptomatic gallstones were selected for Ambulatory LC. They were admitted electively on the same day and operated on in the morning hours and discharged after a check by the surgeon 6–8 hrs later.

**RESULTS:**

Of fifty (*n* = 50) patients selected for ambulatory LC, 92% were discharged successfully after 6–8 hrs observation. No significant perioperative complications were noted. Unplanned admission and readmission rate was 8 and 2%, respectively. Cost saving for the daycare surgery was Rs. 6,200, Rs. 13,300, and Rs.22,800 per patient as compared to in patient general, semiprivate, and private ward package, respectively.

**CONCLUSION:**

Practice ambulatory LC is safe and cost-effective in selected patients with uncomplicated symptomatic gallstones.

## INTRODUCTION

Ability to provide high quality and cost effective care has made ambulatory surgery one of the fastest growing area in the Health Care System all over the world. Primary aim of day care surgery is to provide convenience to the patients by avoiding hospitalization, but patient's safety is the ultimate priority.[1] Rapid recovery after laparoscopic cholecystectomy (LC) and increasing experience with its postoperative course has led to progressively shorter post operative stays and recent trend of true ambulatory LC, without an overnight admission. Reddick and Olsen first reported ambulatory LC in 1990.[2] The safety of ambulatory LC has been demonstrated through its increased use.[3,4] However, practice of its use in our hospital settings is uncommon. A surgical day-care center is well established in our university hospital.

The purpose of present study was to evaluate safety and cost effectiveness of ambulatory LC. Besides the advantages of cost containment results of this study would also help us in substantial reduction of healthcare resources.

## MATERIALS AND METHODS

The study was done at Aga Khan University hospital with total of 480 LCs performed during the period of June 2005 to December 2006 done by eight operating general surgeons. In the present study only three operating surgeons participated. Patients presented at surgical out patient's clinic were evaluated by detailed history and complete physical examination. Those who are having sign and symptoms suggestive of symptomatic gall stone were subjected to laboratory (complete blood count, liver function tests) and radiological workup (ultrasound abdomen). Patients presented to the surgical clinic or casualty with acute episode were not included in the study. Fifty patients fulfilled the selection criteria of uncomplicated symptomatic gallstones. Patients who had given informed consent after full explanation of daycare surgery process were electively admitted for ambulatory LC. Patient's data (demographic, disease, treatment, outcome, and follow-up) was collected on pre-designed computer-based proforma at clinic, day care ward and during follow-up visits.

### Selection criteria

#### Inclusion criteria

Uncomplicated symptomatic cholilithiasis:

1. Sign and symptoms suggestive of symptomatic gall stone
2. Absence of clinical and radiological findings of acute cholecystitis, biliary pancreatitis or empyma gall bladder at the time of admission
3. ASA I-II

#### Exclusion criteria

Those patients having multiple comorbids, coagulation disorder, adverse anesthetic history, other procedures performed along with cholecystectomy or underwent simultaneous bile duct exploration, peroperative finding cholecystitis or suspected/proven malignancy, ASA III or IV and unavailability of competent adult to accompany the patient were excluded from study.

#### Anesthesia protocol

All patients were sent to anesthesia clinic for general anesthesia evaluation. General anesthesia established using following protocol.

***Pre-medication*:** Tablet Midazolam 7.5 mg 1 h before surgery.

***Induction and intubation*:** All patients were given parenteral anti-emetics (Metoclopromide 10 mg) during the induction of anesthesia, Fentanyl 2 microgram/kg, Propofol 1.5-2.5 mg/kg, and Atracurium 0.5 mg/kg.

***Maintenance:*** O_2_ N_2_O Isoflurane, Atracurium boluses as required and Fentanyl repeated 25–50 microgram boluses as required.

### Operative technique

Operation was usually scheduled in the morning session of operating list. Patients were admitted on operative day after midnight fast. All operations were performed by residents supervised by consultant. All patients received single dose of prophylactic antibiotic (Cefazolin 1 gm). The operative field were prepared and draped. Three port technique was used. One port was placed through an umbilical incision and 5 mm trocar (Endoskope®; Karl Storz Tuttlingen, Germany) was placed into the abdominal cavity, 15-mmHg pneumoperitoneum was created and 5 mm O-degree rod lens laparoscope (Karl Storz) was inserted through umbilical port.

After the abdominal survey, the patient was placed in the reverse trendelenburg (Fowler's) position with the patient tilted to the left and the surgeon standing on the patients left side. Two 5 mm ports were placed in subxiphoid (working port) and right upper quadrant (for retraction), respectively. Gall bladder was grasped with one 5 mm clamp at infundibulum and retracted cephaled for linear retraction, and to open up the triangle of Calot's for dissection. Clipping of cystic duct and artery was performed by 5 mm endoclip (Endoskope®; Karl Storz) Peroperative cholangiogram (POC) was performed in selected patients with dilated common bile duct CBD on U/S or deranged liver function tests LFT'S. Gall bladder was removed through umbilical port using endobag. All port sites were injected with 0.5% Bupivacaine local anesthesia at the end of operation.

### Postoperative course

#### Postoperative assessment

Patients were given antiemetic (Ondansetron 8 mg) and I/V NSAIDS (Ketorolac 30 mg) in the recovery room or daycare ward after assessment of verbal pain score (1–10) and assessment of nausea and vomiting by asking about the symptoms by the surgical resident.

#### Admission criteria

Patients were admitted if who had conversion to open cholecystectomy, patient's preference, persistent of nausea or vomiting, persistent pain, drains placement due to extensive dissection.

#### Discharge criteria

Patients were encouraged to get up 4–6 h after surgery and to take a liquid diet and were discharged from daycare unit in the evening once they have adequate pain control, passed urine, and resumed oral feeding. Discharge analgesia included compound analgesic tablets, acetaminophen 325 mg, and oxycodon HCL 5 mg for 3 days.

Patients were instructed and to report to the casualty and also given on call residents and surgical ward reception phone number to contact in case of postoperative problem at home. Two patients reported on phone regarding progress of diet and one reported in the causality on fourth postoperative day due to fever and was admitted.

### Follow up

All patients were followed at surgical clinic after one, two, and four weeks.

### Data analysis

Data analysis was performed with the program Statistical Package for Social Sciences (SPSS, Inc., Chicago, Illinois, USA). Descriptive statistics of numerical variables such as age, rate of conversion to traditional LC or open surgery, mean operative time, admission and readmission rate was computed as mean and ± standard deviation. Frequency and percentage of categorical variables such as reason of conversion, perioperative complications causes of admission and readmission was computed and chi-square test and Fisher's exact test were applied with statistical significant difference in *P*<0.05. Average cost of ambulatory mini LC per case was compared with inpatient package cost of traditional LC.

## RESULTS

**Figure 1:**
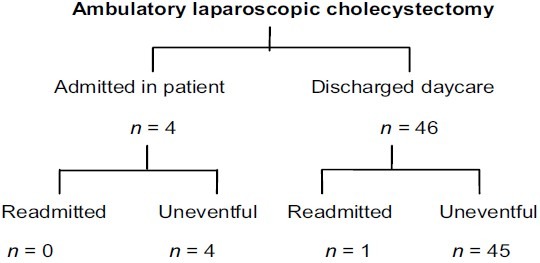
Fate of patients after ambulatory LC

A total of fifty (*n* = 50) patients were selected for ambulatory laparoscopic cholecystectomy (AMLC) according to the selection criteria. Patients demographic data and presenting symptoms are given in Table 1. Operative procedure and peroperative findings are given in Table 2. Mean operative time for LC only was 59 ± 17.1 SD min. Two patients (4%) required conversion to open LC. Obscure anatomy at calot's triangle was the only statistical significant (*P*=0.001) factor contributing to open conversion. Bile/stone spillage occurred in 2/4% patients was irrigated and spilled stones were removed. Perioperative complications (8%) observed are given in Table 3. No statistically significant association was observed in patients having perioperative complications with comorbidities, ASA level, diagnosis at admission, ultrasound findings, white blood count, perioperative finding, and operative time. Mean doses of postoperative analgesia was 1.6 ± 0.69 SD doses. Twenty percent (10/50) of patient's experienced postoperative nausea and vomiting received single dose of ondensetron during postoperative stay, but only one require admission. Forty six (92%) patients were discharged successfully after 6–8 hrs observations in daycare ward. Of remaining four patients, two were admitted because of open conversion, one patient for excessive nausea and vomiting and one admitted because of patient's preference [Figure 1]. Open conversion was found statistical significant factor for unplanned admission (*P*=0.001). Unplanned admission and readmission rate was 8 and 2%, respectively [Table 4]. One patient was readmitted because of postoperative fever on fourth postoperative day and workup revealed small subphrenic collection and patient was discharged after three days conservative treatment with intravenous antibiotics. No statistical significant contributing factor for readmission was observed. Total cost saving for the daycare surgery was Rs. 6,200, Rs. 13,300, and Rs. 22,800/patient as compared to in patient general, semiprivate, and private ward package, respectively [Table 5].

**Table 1:** Demographic data and presenting symptoms

Demographic data *n* = 50	No. (%)
Age	
Mean ± SD	43 ± 13.3
Range	25–60
Sex	45 (90)
Female	5 (10)
Male	30 (60)
ASA	20 (40)
ASA- I	46 (92)
ASA- II	2 (4)
Presenting symptoms	2 (4)
Recurrent biliary pain	50 (100)
Previous cholecystitis	
Previous biliary pancreatitis	
Admission	
Elective	

**Table 2:** Operative procedure and perioperative findings

Perioperative data *n* = 50	No. (%)
Operative procedure	
Mini-lap. Cholecystectomy	44 (88)
Mini-lap. Cholecystectomy and POC	6 (12)
Peroperative diagnosis	46 (92)
Uncomplicated GB	4 (8)
Mucocele conversion	0(0)
Traditional lap. cholecystectomy	2 (4)
Open cholecystectomy	2 (4)
Reason for conversion	
Obscure anatomy	50
Histopathology	(100)
Chronic cholecystitis	

**Table 3:** Perioperative complications

Perioperative complications *n* = 50	No. (%)
Port site bleeding	0
Bile/stone spillage	2 (4)
Cystic artery bleeding	1 (2)
Sub hepatic collection	1 (2)
Other vascular injury	0
CBD injury	0
Bowel injury	0
Pancreatitis	0

**Table 4:** Unplanned admission and readmission rate

Unplanned admission and readmissions *n* = 50	No (%)
Unplanned admissions	
Open conversion	2 (4)
Nausea and vomiting	1 (2)
Patients preference	1 (2)
Readmissions	
Postoperative fever	1 (2)

**Table 5:** Inpatient traditional LC vs. daycare ambulatory LC cost comparisons and saving

Inpatient (traditional LC)	Daycare (ambulatory LC)	Cost saving
Stay Length Package price (per case)	Stay Length Average price (per case)	
Ward 1-2 days 31,200	Daycare 6-8 hrs 25,000	6,200
Semiprivate 1-2 days 38,300	Daycare 6-8 hrs 25,000	13,300
Private 1-2 days 47,800	Daycare 6-8 hrs 25,000	22,800

## DISCUSSION

With growing economic pressure, some centers have advocated routine application of ambulatory LC in all patients with no pre-selection criteria.[5–8] However, day surgery involves a shift of postoperative care from hospital to the patient after discharge. Recruitment of high risk patients presents a challenge to the limits of safe practice, particularly during the early postoperative day. Mortality has been reported after ambulatory LC.[9] Performance of ambulatory LC in high risk patients requires scrupulous evaluation prior to implementation.[10,11] A crucial aspect in the development of safe ambulatory surgery program is the criteria for patient's selection. Robinsons *et al*[12] reported their experience in a public academic institution have achieved outpatient LC in 70% of an unselected patients and they have identified ASA classification, procedural duration and surgery start time as factor associated with failure of outpatient management. Some authors have come to the conclusion that appropriate patients selection lowers failure rate and patients most likely to fulfill the criteria of outpatient LC, who have an anesthetic preoperative classification of ASA grade I or II, with no previous abdominal surgery no history of acute cholecystitis and a procedural duration of shorter than 90 min.[2,9,13] Most studies utilize selection criteria when evaluating patients for outpatient LC.[14,15] We preferred to offer day care surgery to selected patients while evaluating in outpatient clinic based on selection criteria (Elective Ambulatory LC for symptomatic cholelithiasis, absence of clinical and radiological evidence of acute cholecystitis at admission, ASA-I, II and selected patients with ASA-III) resulted in successful adaptation of AMLC in 92% of patients. The rate of unplanned admission in ambulatory LC is a quality index as it might represent the existence of inadequate criteria in selection of patients who given their characteristics, precedents, or preoperative findings were not candidate to this type of surgery. This percentage in analyzed series is 1–39%. A lower admission rate has been reported in freestanding ambulatory surgery centers and this could be related to their strict patient's selection criteria.[13,14] Our unplanned admission rate of 8% in present study compares favorably with the results of other centres in appropriately selected patients.

A number of factors preclude ambulatory LC including development of undesirable postoperative symptoms (abdominal postoperative pain, nausea or vomiting), length of operative procedure, open conversion, delayed detection of potential postoperative complications and discharge acceptance by the patient.

Postoperative nausea and vomiting remained a frequent reason for unplanned admission after ambulatory LC.[16] Several authors recommend the use of standard protocols to minimize postoperative symptoms of pain, nausea or vomiting. Methods used to prevent nausea include avoiding the use of volatile anesthetic agents and the undue use of opiates in the postoperative period. Ondensetron and cyclizine were chose as effective antiemetic in reducing postoperative nausea or vomiting.[17,18] Optimal control of postoperative pain, nausea or vomiting is pivotal to enhancing the outcome of ambulatory LC. In present study, use of ondensetron, nonsteroidal anti-inflammatory medication instead of opiates in patients experienced postoperative nausea or vomiting might have attributed to our less unplanned admission rate due to nausea and vomiting. From the surgeon's prospective complete deflation of the peritoneal cavity by releasing carbon dioxide after cholecystectomy may be a useful adjunct in reducing postoperative nausea or vomiting. It seems reasonable to assume that a reduction in the size of ports used in elective minimal access LC sustain or enhance the postoperative recovery and thus performing ambulatory LC. In our study, we used three ports, 5 mm instruments along with local administration of Bupivacain in elective AMLC may had attributed to minimal postoperative analgesic requirement and hence none of our patient admitted for postoperative pain.

Admission is usually a joint decision made by the surgeon and the patient. As patient has to participate in self care after discharge, their comfort, preference, and safety need to be considered on the assessment for discharge. One of our patients admitted of his preference because he was uncomfortable to be discharged. Two (4%) had open conversion and hence required admission. Like other studies,[19] the reason for open conversion in present study was obscure anatomy due to aberrant right hepatic artery.

Length of operation has been shown to be the most important factor for unplanned admission after ambulatory LC.[20,21] This current study performed at a teaching hospital, where surgical trainees are an integral part of patient care, virtually all operation were performed by residents under consultants supervision. The current length of operation with a mean of less than 60 min was encouraging. This suggests that ambulatory LC can be an integral part of training program for surgical resident without compromising patient care and outcome.

Diverse authors question the safety of ambulatorization arguing the potential for delayed detection and therefore solution of postoperative complications that cannot be demonstrated within 24 hrs of surgery.[9,22] The main reticence regarding the ambulatory LC approach derives from the fact that many surgeons prefer period of at least 24 hrs with overnight stay which avoids delay in detection of some complication in the immediate postoperative period. On the contrary, other authors argue that the incidence of life threatening complication including arterial bleeding is very low 1:2000 and as such is symptomatic within hours during the postoperative period thus being possibly detected while the patient is still in hospital.[23] A complication such as biliary iatrogenic develops in LC between 0.3 and 1% of cases.[24] If not detected intraoperatively, secondary symptoms develops including a biliary collection (abdominal pain, fever, etc.) or jaundice during the postoperative period. These complications cannot be detected before the second postoperative day. In our case, one of our patients developed hemorrhage from cystic artery which was detected intraoperatively and homeostasis was achieved. One patient (1/2%) was readmitted with small subphrenic collection. This was detected with postoperative fever, on fourth postoperative day, which was resolved with intravenous antibiotic without need for per-cutaneous aspiration. Likewise, in our study, the selection of patients with elective uncomplicated symptomatic cholelithiasis (absence of clinical and radiological findings of acute cholecystitis) contributed to reduce cases with likely postoperative complications (biliary injury). No successfully discharged patient had a major postoperative complication. Therefore, a period of prudent observation for 6–8 hrs may suffice, due to the fact that overnight would not reduce the detection of subsequent major complications.

Average total cost was different for the AMLC as compared to inpatient package cases. The main cost saving were in room costs (general ward, semi private or private) and institutional data, however, operative procedure cost was similar in both cases.

Our results demonstrated that ambulatory LC as an outpatient day care procedure is safe with high success rate in carefully selected patients with uncomplicated symptomatic gallstones and has the advantage of cost effectiveness. In these selected patients, three ports technique may reduce the postoperative pain and can further enhance early recovery.

## CONCLUSION

Practice AMLC is safe in carefully selected patients and has the advantage of cost effectiveness. This will help us in reducing healthcare costs, use of hospital beds and thus waiting time.
